# Evaluation of the effects of sequence length and microsatellite instability on single-guide RNA activity and specificity

**DOI:** 10.7150/ijbs.37152

**Published:** 2019-10-03

**Authors:** Changzhi Zhao, Yunlong Wang, Xiongwei Nie, Xiaosong Han, Hailong Liu, Guanglei Li, Gaojuan Yang, Jinxue Ruan, Yunlong Ma, Xinyun Li, Huijun Cheng, Shuhong Zhao, Yaping Fang, Shengsong Xie

**Affiliations:** 1 Key Laboratory of Agricultural Animal Genetics, Breeding and Reproduction of Ministry of Education & Key Lab of Swine Genetics and Breeding of Ministry of Agriculture and Rural Affairs, Huazhong Agricultural University, Wuhan 430070, P. R. China;; 2 Agricultural Bioinformatics Key Laboratory of Hubei Province, Hubei Engineering Technology Research Center of Agricultural Big Data, College of Informatics, Huazhong Agricultural University, Wuhan 430070, P. R. China;; 3 The Cooperative Innovation Center for Sustainable Pig Production, Huazhong Agricultural University, Wuhan 430070, P. R. China.

**Keywords:** CRISPR/Cas9, length, microsatellite, activity, specificity

## Abstract

Clustered regularly interspaced short palindromic repeats (CRISPR)/Cas9 technology is effective for genome editing and now widely used in life science research. However, the key factors determining its editing efficiency and off-target cleavage activity for single-guide RNA (sgRNA) are poorly documented. Here, we systematically evaluated the effects of sgRNA length on genome editing efficiency and specificity. Results showed that sgRNA 5′-end lengths can alter genome editing activity. Although the number of predicted off-target sites significantly increased after sgRNA length truncation, sgRNAs with different lengths were highly specific. Because only a few predicted off-targets had detectable cleavage activity as determined by Target capture sequencing (TargetSeq). Interestingly, > 20% of the predicted off-targets contained microsatellites for selected sgRNAs targeting the dystrophin gene, which can produce genomic instability and interfere with accurate assessment of off-target cleavage activity. We found that sgRNA activity and specificity can be sensitively detected by TargetSeq in combination with *in silico* prediction. Checking whether the on- and off-targets contain microsatellites is necessary to improve the accuracy of analyzing the efficiency of genome editing. Our research provides new features and novel strategies for the accurate assessment of CRISPR sgRNA activity and specificity.

## INTRODUCTION

Clustered regularly interspaced short palindromic repeats (CRISPR)/Cas9 technology-mediated genome editing enables site-specific knockout, insertion, and base editing of mammalian cells 1-5. This method is faster, cheaper, more accurate, and more efficient than traditional genome editing technology and has a wide range of potential applications 6. This technology is also attractive for the development of novel therapies. For instance, Leber congenital amaurosis type 10 (LCA10) is a severe retinal dystrophy caused by mutations in *CEP290* gene 7. Several studies performed CRISPR-mediated repair of *CEP290* gene mutation sites 8, 9. In particular, a previous work showed that the development of EDIT-101 for LCA10 and additional CRISPR-based medicines can be used for the drug treatment of inherited retinal disorders 10. Duchenne's muscular dystrophy is caused by mutations in the dystrophin* (DMD)* gene, which encodes a protein necessary for muscular contraction 11, 12. CRISPR/Cas9 technology poses an attractive platform for *DMD* gene therapy and can be used to repair mutations or mediate exon skipping 13, 14. Although this technology can be efficiently implemented for genome modification, one of the most important issues is its potential off-target effect. Double-strand breaks (DSBs) can be generated in locations outside the intended site and can introduce unexpected mutations that need to be carefully monitored, particularly when using these tools for therapeutic purposes. However, some controversies about the off-target effect of CRISPR/Cas9 still exist. High-frequency off-target mutagenesis can be induced by CRISPR/Cas9 strategy in human or mouse cells 15-17. In genetically edited mice, goats, and cotton plants, CRISPR/Cas9-mediated genome editing has a low incidence or undetectable genome-wide off-target mutations 18-21.The factors that determine editing efficiency and off-target cleavage activity for single-guide RNA (sgRNA) must be identified to improve the reliability of CRISPR/Cas9-based genome editing for therapeutic applications.

Many factors, such as nucleotide (nt) composition and secondary structure 22, 23, DNA supercoiling on adjacent DNA sites 24, heterochromatin 25, chromatin state and structure 26, 27, and nucleosome positioning 28, can affect the efficiency and accuracy of CRISPR/Cas9. A previous work showed that two short sequence motifs at the 3′-end of the targeting sequence can inhibit sgRNA activity 29. In particular, the length of 5′-end sequence is heavily associated with sgRNA activity and specificity. However, some controversies about the benefits of changing sgRNA length for genome editing experiments exist. Truncated guide RNAs (tru-gRNAs) with short regions of the target complementarity, that is, < 20 nts in length, can improve CRISPR/Cas9 nuclease specificity without sacrificing on-target genome editing efficiencies 30, 31. A previous study showed that tru-gRNAs with 17-19 nts spacer are more sensitive to mismatches than those with length of 20 nts, which can effectively reduce off-target mutations 30. Meanwhile, tru-gRNAs with 18 complementary nts and Cas9 nucleases can effectively generate gene knockout mice with a significantly high efficiency in a site-dependent manner 32. However, the use of tru-gRNAs can reduce CRISPR/Cas9-mediated genome editing activity in a cell type-dependent manner 33, 34. Cas9 with a 17-nt sgRNA has lower on-target affinity and reduced editing efficiency compared with Cas9 with a full-length (20 nts) sgRNA. Further truncation of sgRNA to 15 nts can reduce DNA-binding affinity and fully abolish on-target cleavage 34. Therefore, the effect of 5′-end lengths on sgRNA activity and specificity should be further studied especially when using CRISPR/Cas9 technology for gene therapy.

In a CRISPR/Cas9 experiment, CRISPR can still cause inadvertent changes to the genome even with optimized sgRNA or nucleases. Generally, two methods can detect these effects. The first method is the detection of these effects based on further experimental methods, such as T7 endonuclease I (T7ENI) cleavage and restriction enzyme assays, and the second method is the prediction of CRISPR's off-target activity using computational algorithms that identify possible off-target sites based on sgRNA sequence 35, 36. T7ENI cleavage assay is the most widely used experimental method 2 and detects heteroduplex DNA, including desired mutations with a wild-type DNA strand, that results from annealing a DNA strand. Restriction enzyme assay is another frequently used experimental method to evaluate Cas9 activity when a target region contains a suitable restriction enzyme site. *Escherichia coli* TOPO TA subcloning and Sanger sequencing methods can also be adopted. CRISPR/Cas9 genotyping can be used for restriction fragment length polymorphism assay 37. Polymerase chain reaction (PCR)-based protocol and high-resolution melt analysis were also developed to detect insertion-deletion (Indel) mutations and to assess on- and off-target efficiencies, respectively 38, 39. Researchers have developed multiple *in vitro* and cell-based techniques to detect CRISPR off-target mutations in an unbiased and genome-wide manner to overcome the limitations of *in silico* prediction 40, 41. These methods are critical in developing therapeutic approaches, because they can detect rare and unpredictable off-target sites that can have potentially harmful effects on a patient. However, the use of high-throughput sequencing strategies to assess off-target effects remains controversial. For instance, considerable uncertainties exist at the genomic level, such as genomic instability caused by microsatellite instability and chromosome instability, which can lead to unpredictable DSB and can interfere with the accurate assessment of off-target effects 42, 43. Therefore, the effects of genomic instability on sgRNA activity and off-target cleavage rate need to be elaborated. The absence of an effective algorithm is also a problem in fast and accurate CRISPR high-throughput data analysis. Several online or stand-alone tools, such as Cas-analyzer 44, CrispRVariants 45, and CRISPResso 46, are available for analyzing high-throughput CRISPR sequencing, but these tools are either limited by the network or have slow speed in the calculation for local service, which hinders their further application.

In this study, the effects of 5′-end sequence lengths on sgRNA activity and off-target effects were systematically evaluated, and the sensitivities of different assays in assessing sgRNA activity were compared. In particular, the specificities of sgRNAs with different lengths were compared using Target capture sequencing (TargetSeq) combined with *in silico* prediction. Off-target sites containing microsatellites can interfere with the accurate assessment of off-target cleavage activity. Our study provides novel features to design specific and efficient CRISPR sgRNA and strategies to accurately detect sgRNA activity and off-target effect.

## RESULTS

### Variation in 5′-end lengths of sgRNAs can alter genome editing efficiency

**Figure 1:**
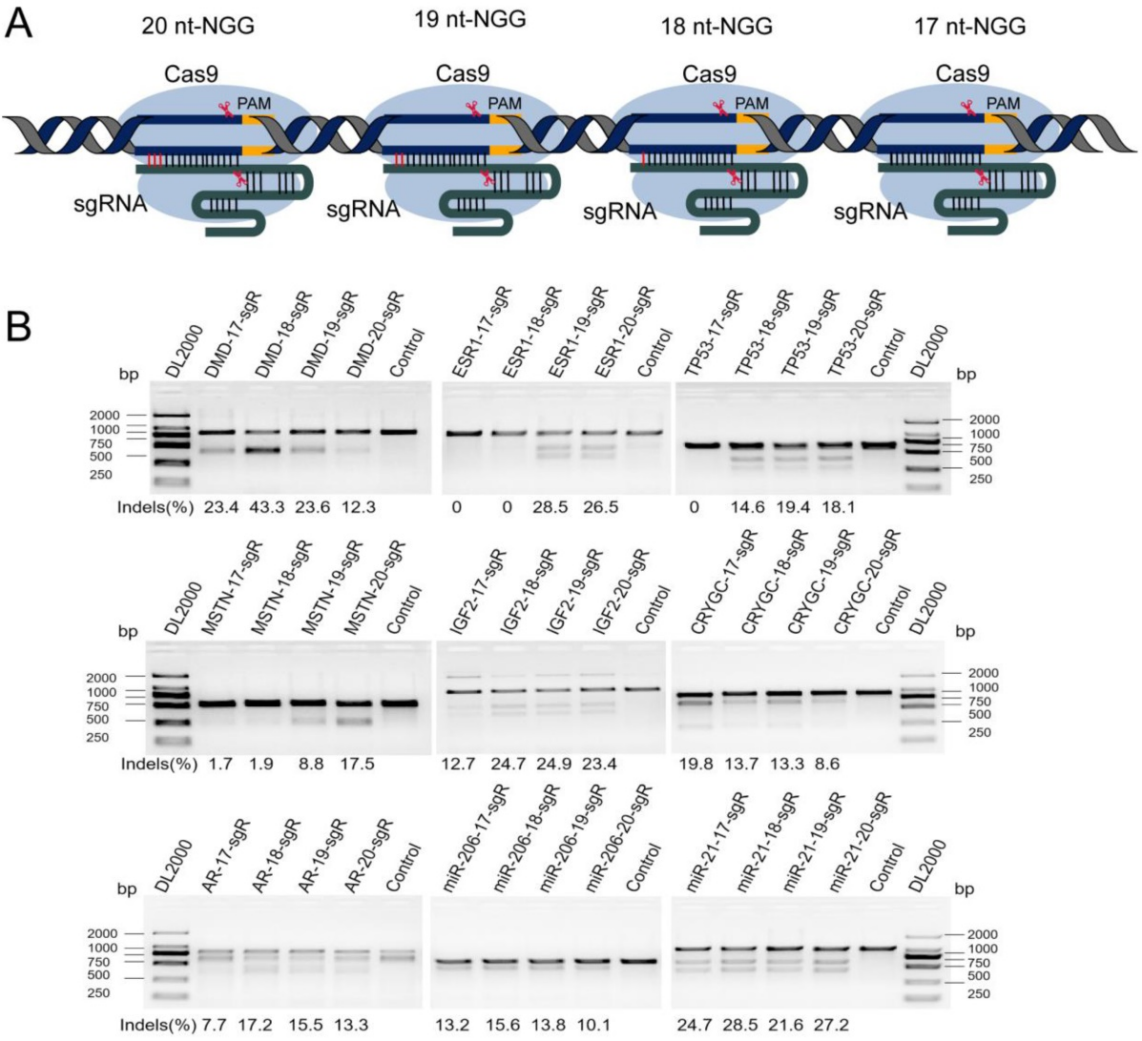
Difference in the activity at the same genomic location in different lengths of sgRNAs. (A) Scheme of using 20, 19, 18, 17 nts of sgRNAs on target genes. The sequence patterns are recognized including N_20_NGG, N_19_NGG, N_18_NGG, and N_17_NGG. (B) Activities of sgRNAs in different lengths on target genes using T7ENI cleavage assay. “NGG” represents protospacer adjacent motif (PAM) sequences, N represents one of four bases, including adenine (A), guanine (G), cytosine (C), and thymine (T); sgR: single-guide RNAs; bp: base pairs; DL2000:DNA marker, control: wild-type control cells.

Seven protein encoding genes, including *DMD*, estrogen receptor 1 (ESR1), tumor protein p53 (TP53), myostatin (MSTN), Insulin-like growth factor 2 (IGF2), Crystallin Gamma C (CRYGC), Androgen receptor (AR), and two microRNAs (miRNAs), namely, miR-206, and miR-21, were randomly selected to assess the effect of 5′-end lengths on sgRNA activity. Subsequently, sgRNAs with full-length (20 nts) and those truncated to 19, 18, and 17 nts were designed by CRISPR-offinder (Figure 1A). The sgRNA expression plasmid was co-transfected into human embryonic kidney cell line 293T (HEK293T) cells with Cas9 expression plasmid. After extracting cellular genomic DNA (gDNA), sgRNA activity was detected by T7ENI cleavage assay. Results showed that truncated sgRNAs with lengths of 17 and 18 nts had no cleavage activity when targeting the *ESR1* gene (Figure 1B). Meanwhile, the truncated sgRNA with length of 17 nts had no cleavage activity when targeting the *TP53* gene (Figure 1B). When targeting the *MSTN* gene, sgRNA activity showed a 10-fold decrease (from 17.5% to 1.7%) after length truncation (Figure 1B). By contrast, the activity of different lengths of sgRNAs targeting the same genome site of miR-21 and miR-206 is almost unchanged (Figure 1B). Subsequently, the activities of full-length and truncated sgRNAs were further validated by amplicon high-throughput sequencing, and sequencing data were analyzed by CRISPRamplicon. As shown in Figure S1, the activity trends of different lengths of sgRNAs detected by Amplicon Sequencing (AmpliconSeq) and T7ENI cleavage assay were almost the same for most target sites, indicating that the cleavage activities of these sgRNAs were accurately assessed. These results showed that the 5′-end lengths of sgRNA can affect the cleavage activity of genome editing. Thus, by simply altering the 5′-end lengths of sgRNA, the Cas9 nuclease can be guided to the same sites in the genome, but the cleavage activity of CRISPR/Cas9-mediated gene knockout will change.

### Potential off-target sites of sgRNA significantly increased after 5′-end nucleotide truncation

**Figure 2:**
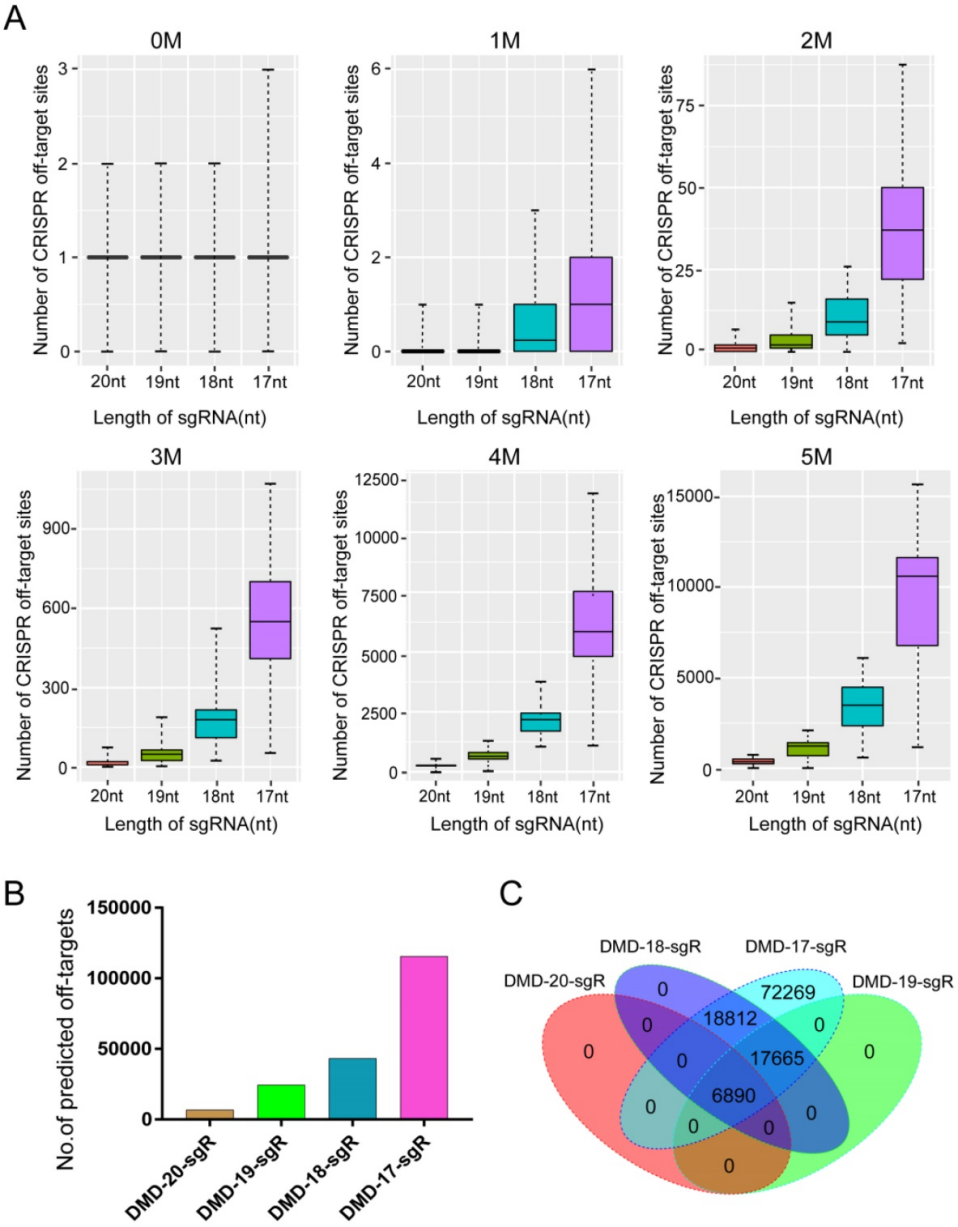
Predicted specificity of sgRNAs in different lengths targeting *DMD* gene. (A) Differences in the predicted number of off-target sites with 1, 2, 3, 4, 5 nucleotide mismatches. T-test was performed on the total predicted off-target sites of sgRNAs with 19, 18, 17 nts against 20 nt in length. *P* values are 1.5e-11, 3.17e-16, 9.87e-17, respectively. (B) Difference in the predicted total number of off-target sites of 20, 19, 18, 17 nt sgRNAs. (C) Venn diagram of the predicted off-target sites in different lengths of sgRNAs. nt: nucleotides; sgR: small guide RNA; M representsthe number of nucleotide mismatches (1M, 2M, 3M, 4M, or 5M); 0M represents the perfect match to the on-target site; off-target sites are counted at the number of 0M sites > 1.

The potential off-targets of sgRNA with different lengths were predicted to assess the effect of sgRNA 5′-end lengths on genome editing specificity. The *DMD* gene was selected as candidate target. The lengths of sgRNA and protospacer adjacent motif (PAM) sequence were set as follows: on-target: 20, 19, 18, or 17 nts + NGG (N = A, T, C, or G); and off-target: 20, 19, 18, or 17 nts + NRG (R = G or A). As a result, 162 sgRNAs with lengths of 20, 19, 18, and 17 nts targeting the *DMD* gene were designed, and > 100,000 potential off-target sites that differed from the sgRNA by up to five mismatches in the genome were predicted. As shown in Figure 2A, when sgRNA length was truncated from 20 nts to 17 nts, the number of potential off-target sites that exactly match the genome increased slightly. Subsequently, T-test was performed on the total predicted off-target sites of sgRNAs with lengths of 19, 18, and 17 nts against 20-nt sgRNAs. The calculated *P* values of 19-, 18-, and 17-nt sgRNAs were 1.5e-11, 3.17e-16, 9.87e-17, respectively (Figure 2A). This result showed that the number of mismatches between the potential off-target site and sgRNA increased from 1 to 5, and the number of predicted off-target sites increased significantly. For instance, after analyzing potential off-target sites of sgRNAs with 20, 19, 18, and 17 nts targeting the same site of the *DMD* gene, the result showed that if up to 5 mismatches are present between sgRNA and the potential off-target site, tens of hundreds of potential off-target sites are predicted (Figure 2B). As shown in Figure 2B, when the length of sgRNA targeting *DMD* was truncated to 17 nts, the total number of predicted off-targets reached 115,636. Subsequently, according to Venn diagram analysis, the number of potential off-target sites overlapping among 20-, 19-, 18-, and 17-nt sgRNAs was 6,890 (Figure 2C), indicating that as the length of the candidate sgRNA shortens by 1 nt at a time, a considerable number of new potential off-target sites are produced (Figure 2C). Furthermore, 10,404 sgRNAs with lengths of 20, 19, 18, and 17 nts targeting 100 randomly selected human protein-coding genes were designed, and 76,073,885 potential off-target sites were predicted using CRISPR-offinder (Figure S2). This result showed that if the number of mismatches between potential off-target sites and sgRNA increased, many predicted off-target sites for truncated sgRNAs were found (Figure S2).

### AmpliconSeq and TargetSeq sequencing strategy allowed highly sensitive detection of sgRNA activity

**Figure 3:**
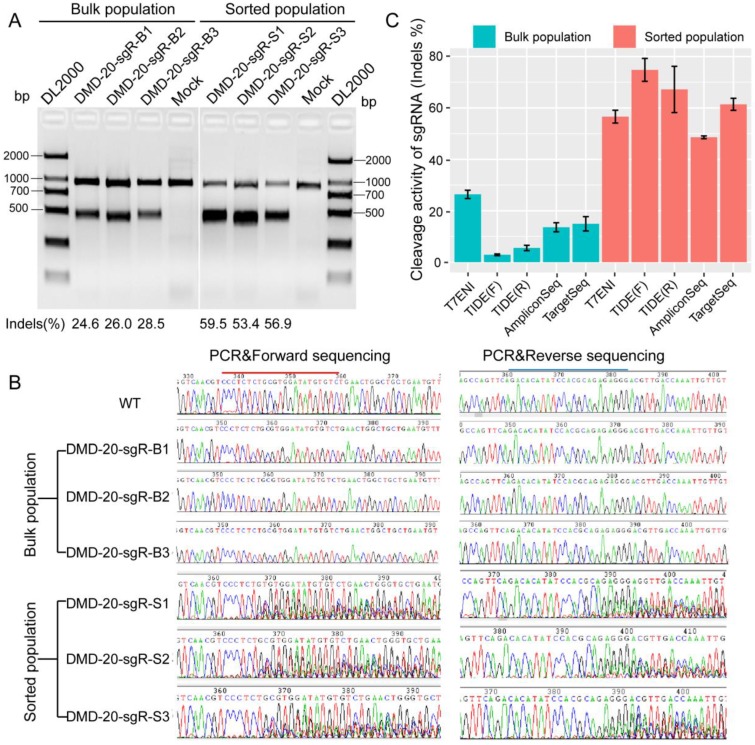
The sensitivities of different methods in detecting the activity of 20 nt-long sgRNA. (A) The activity of sgRNA on targeting *DMD* gene by T7ENI cleavage assay. (B) PCR products were sequenced in both directions. (C) Assessing sgRNA activity by four methods. These methods includes T7ENI cleavage assay; Sanger DNA sequencing followed by TIDE web-based software analysis; High-throughput amplicon sequencing (AmpliconSeq); and Target capture sequencing (TargetSeq). Bulk population represents unsorted cells; Sorted population represents sorted cells; Mock control represents Lipofectamine 2000 only; WT: wild-type cells; TIDE(F): Sanger DNA forward-sequencing data; and TIDE(R): Sanger DNA reverse-sequencing data.

The performance of six methods in detecting genome editing activity was evaluated to select the most suitable method for high-throughput assessment of activity and specificity of sgRNA with different lengths. The methods evaluated included T7ENI cleavage assay; Sanger sequencing of PCR products; *E. coli* TOPO TA cloning and Sanger sequencing; Sanger DNA sequencing of PCR products, followed by TIDE web-based software analysis (https://tide.nki.nl); high-throughput AmpliconSeq; and TargetSeq. The high-throughput sequencing results of AmpliconSeq and TargetSeq were analyzed by CRISPRamplicon. A fluorescence-activated cell sorting (FACS)-based sorting strategy, which can be used to enrich and select genetically modified cells, was adopted. Enriched or nonenriched genome-edited cells were used as test samples to evaluate the sensitivity of the six methods. As shown in Figure 3A, T7ENI cleavage assay showed that the average editing efficiency of a 20-nt sgRNA, which targeted the *DMD* gene, was 26.37% in nonenriched genome-edited cells. When genome-edited cells were subjected to FACS-based sorting, sgRNA activity increased by approximately two-fold, and the average editing efficiency reached 56.6%. The corresponding genome-edited cells were detected by other methods. For instance, on-target sites were amplified by PCR, and forward and reverse Sanger sequencing were performed separately in PCR products. As shown in Figure 3B, the peak patterns of sequencing traces between nonenriched genome editing and wild-type cells at the sgRNA on-target site were not different, whereas those between enriched genome editing and wild-type cells at the sgRNA on-target site were significantly different. Thus, the cleavage activity of sgRNAs can be easily distinguished by the peak patterns in the electropherograms (Figure 3B).

Subsequently, PCR sequencing results were further analyzed by TIDE software. As shown in Figure 3C, the average cleavage activity of sgRNA was 5.5% in nonenriched genome-edited cells, whereas the average editing efficiency reached 74.1% in enriched genome-edited cells. Thus, compared with the results obtained by T7ENI cleavage assay, the obtained cleavage activity of sgRNA by TIDE software was inconsistent (Figure 3C). Although TOPO TA cloning and Sanger sequencing methods were widely used to identify mutation genotype (Figure S3), genome editing efficiency was difficult to accurately assess because of the low number of randomly selected TA clones. By contrast, AmpliconSeq and TargetSeq methods showed that the average cleavage activities of sgRNAs in nonenriched genome-edited cells were 13.6% and 14.9%, respectively, whereas the average genome editing activities in enriched genome-edited cells were 48.1% and 64.4%, respectively (Figure 3C). These results were comparable to those of T7ENI cleavage assay. Among the six methods, AmpliconSeq and TargetSeq methods were observed to sensitively detect sgRNA activity.

### TargetSeq revealed rare off-target mutations in CRISPR/Cas9-edited cells using sgRNAs with different lengths

sgRNAs with 20, 19, 18, and 17 nts targeting the same genome site of *DMD* gene were selected to further evaluate the specificity of sgRNAs with different lengths (Figure 2B and 2C). A total of 6,549 capture probes for 6,890 candidate off-target sites, which were shared by different sgRNA lengths, were designed and synthesized to perform TargetSeq. Enriched genome-editing cells were prepared by FACS-sorting method, and sgRNA samples were first evaluated by T7ENI cleavage assay. As shown in Figure S4, the average cleavage activities of sgRNAs with 20, 19, 18, and 17 nts were 67.4%, 61.8%, 64.3%, and 72.1%, respectively. Subsequently, nonenriched and enriched genome-editing cells, including wild-type cells, were selected for TargetSeq. Approximately 2.5 GB of clean data for each sample (approximately 300-fold) were obtained. Using custom Perl program, approximately 200 bp candidate target sequences were extracted from the upstream and downstream of on- and off-target sites of sgRNAs and analyzed by CRISPRamplicon. As shown in Figure S5A, the proportions of candidate target sequences were approximately 73% and 5% for number of sequencing reads ≥ 10 and ≤ 10 per sample, respectively. However, approximately 20% of candidate off-target sequences remained uncaptured. Captured target sequences with number of sequencing reads < 10 were discarded to improve high-throughput sequencing quality. Thus, the capture and high-throughput sequencing coverage of 6,890 potential off-target sites of sgRNA were averaged at approximately 70%, with a sequencing depth of up to 300-fold for each sample (Figure S5A). After comparing the CRISPR high-throughput sequencing data with sequencing depths of 300- and 3000-fold, sgRNA activity was detected to be consistent (Figure S6). Results showed that a sequencing depth of approximately 300-fold was suitable in detecting sgRNA activity.

**Figure 4:**
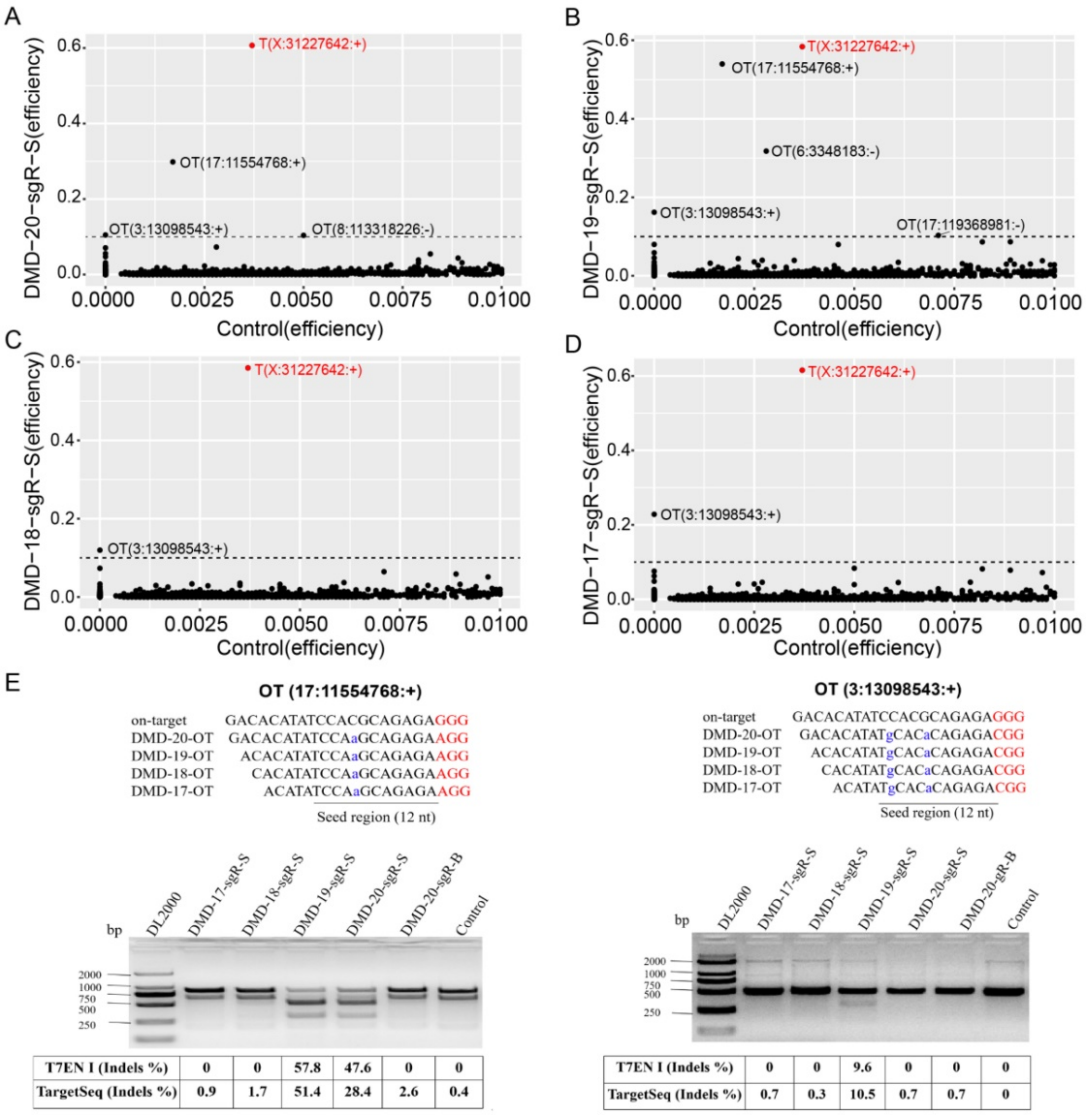
Detection of on- and off-target site cleavage activities in different lengths of sgRNAs but targeting *DMD* gene based on *in silico* prediction and Target capture sequencing. (A), (B), (C), and (D) are the results of the on- and off-target cleavage efficiencies of 20, 19, 18, and 17 nt sgRNAs by Target capture sequencing in the sorted cell population, respectively. T (X:31227642:+) represents the on-target site, while OT for off-target site of sgRNA. The x- and y-axis represent the Indel efficiencies of on- and off-targets for sgRNAs in the control and gene-editing groups, respectively. The number of reads ≧ 10 is the threshold of control group representing the captured target, and the Indel efficiency is ≦ 1 %. (E) Validation of off-target sites for 20, 19, 18, and 17 nt sgRNAs using T7ENI cleavage assay. The Indel efficiency below the agarose gel electrophoresis shows the detection of the same predicted off-target site by the T7ENI cleavage assay and TargetSeq. Seed region represents seed sequences, which are the first 1-12 positions of the spacer immediately in the 5′ end to the PAM sequence. Control represents the negative control group; DL2000: DNA ladder; Nucleotides marked in red and blue colors represent protospacer adjacent motif (PAM), and mismatches and OT for off-target, respectively, OT: predicted off-target site.

For the same target capture site, the threshold of editing efficiency detected in the control group (wild-type cells) was set to 1% to reduce background noise, and editing efficiency ≥ 1% for each captured target site in any genome-modified sample was removed. The control group was used as reference. The genome editing efficiency and predicted off-target sites of sgRNA on-target in different experimental groups were analyzed. As shown in Figure S5B, when genome editing assay was performed using 20-nt sgRNA in nonenriched genome-edited cells, except those in the on-target site, no cleavage activity was detected by TargetSeq in approximately 5,276 predicted off-targets with sequencing reads of up to 10. Meanwhile, in FACS-enriched genome-edited cells, the genome editing efficiencies of three predicted off-target sites were 51.4%, 31.7%, and 10.5% (Figure 4A). When the genome editing assay was performed using a 19-nt sgRNA, four predicted off-target sites were detected to have off-target activities. Among these off-target sites, one had a genome editing efficiency of 51.4% (Figure 4B). When genome editing assay was performed using 18- and 17-nt sgRNAs, only one identical predicted off-target site with relatively low genome editing efficiency was detected (Figure 4C and 4D). Next, two predicted off-target sites were further validated by T7ENI cleavage assay (Figure 4E). Therefore, only 3, 4, 1, and 1 off-target sites were detected by TargetSeq in 20-, 19-, 18-, and 17-nt sgRNAs, respectively.

### Microsatellite located in predicted off-target sites can interfere with accurate assessment of sgRNA specificity

**Figure 5:**
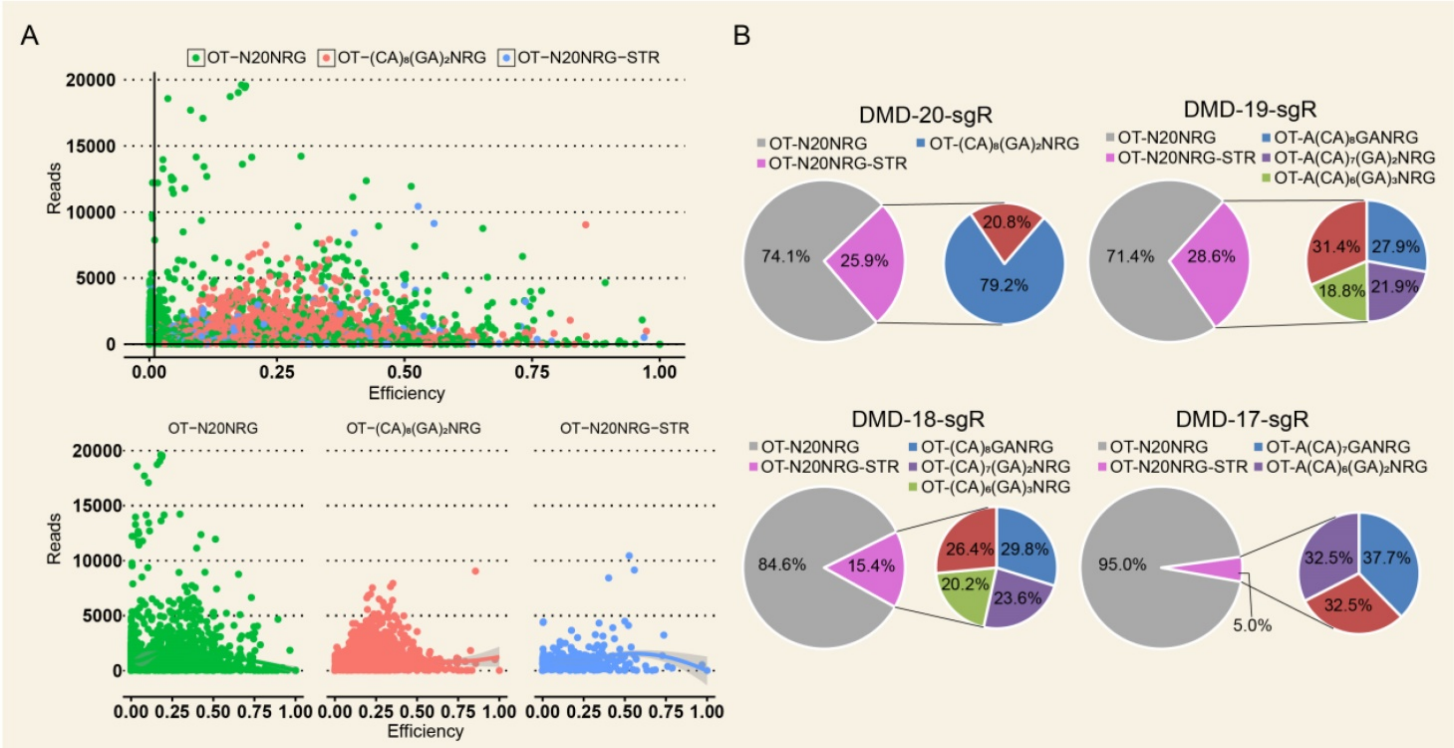
Indel frequency and reads distribution of CRISPR/Cas9 off-target sites containing microsatellites in the control group. (A) Distribution of captured predicted off-target sites with different sequence features in the control group. (B) Distribution of the editing efficiency and reads in predicted off-target sites with different sequence features in the control group. (C) Distribution of the microsatellites in predicted off-target sites in 20, 19, 18, and 17 nt sgRNAs. NRG: protospacer adjacent motif (PAM); N = A, T, C, or G; R = A or G; STR: short tandem repeat; OT: predicted off-target site.

**Figure 6:**
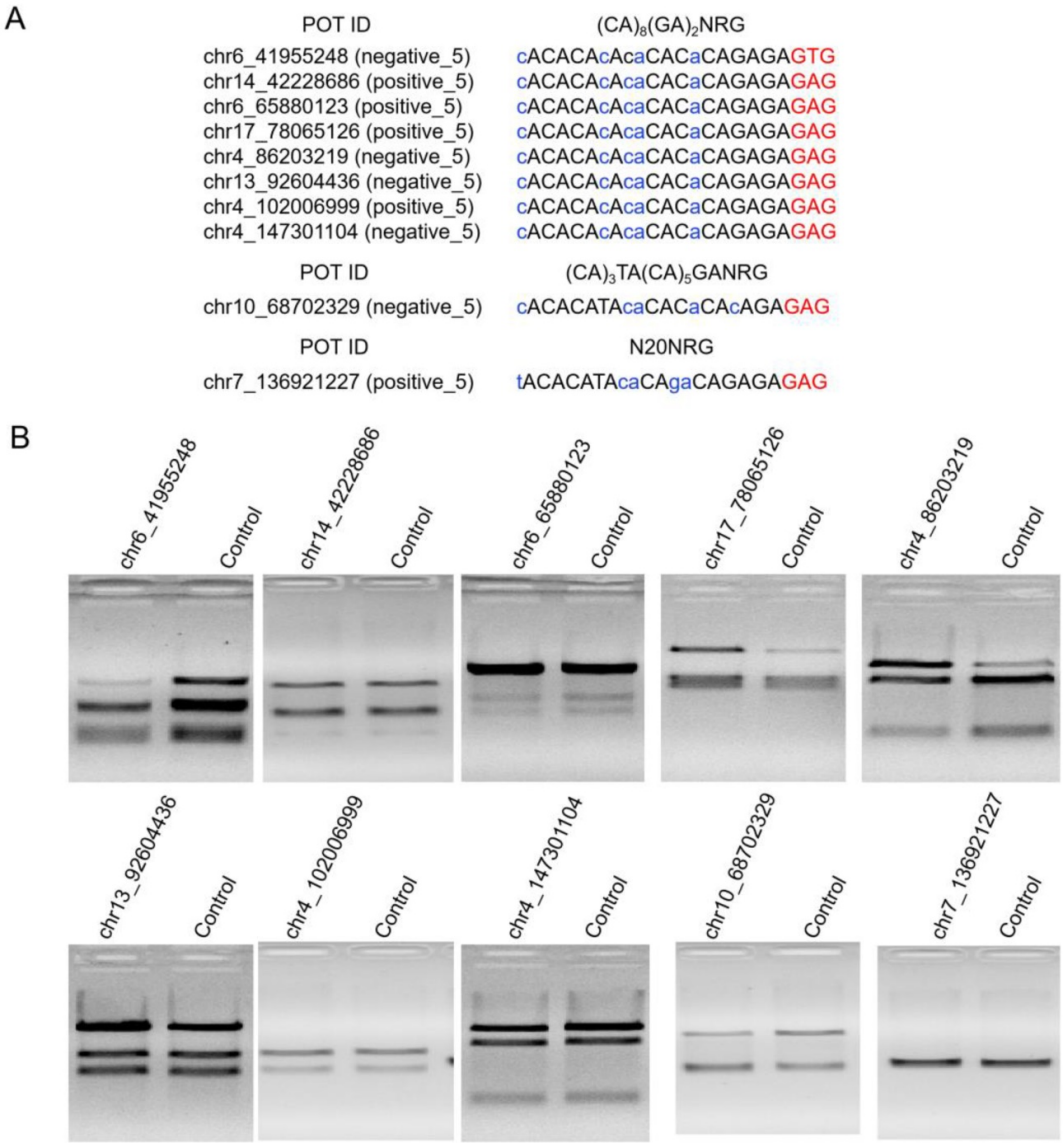
Validation of selected predicted off-target sites containing microsatellites. (A) Sequence and microsatellite features of potential off-target sites. (B) Detection of cleavage activity of the predicted off-target sites by T7ENI cleavage assay. Control represents negative control group. POT: potential off-target site; ID: identity number; NRG: protospacer adjacent motif (PAM); N = A, T, C, or G; R = A or G. Nucleotides marked in red and blue colors represent PAM and mismatches, respectively.

High Indel mutation frequency was found even in the control group when TargetSeq was used. We analyzed the sequence characteristics of predicted off-target sites to identify possible causes. Microsatellites occur in thousands of locations in an organism's genome and have higher mutation rates than other DNA fragments. Thus, the microsatellites of predicted off-target sites of sgRNA with different lengths were analyzed using the MISA software. Results showed that some predicted off-target sites contained microsatellites (Figure 5A). The proportions of microsatellites corresponding to the predicted off-target sites of the selected 20-, 19-, 18-, and 17-nt sgRNAs were 25.9%, 28.6%, 15.4%, and 5%, respectively (Figure 5B). At sgRNA length of 20 nts, the proportion of (CA)_8_(GA)_2_ reached 79.2%. When the length of sgRNA was shortened, this microsatellite type derived three new microsatellites, namely, A(CA)_8_GA, (CA)_8_GA, and A(CA)_7_GA. Subsequently, the distribution of sequencing reads and Indel mutation frequency for the captured predicted off-target sites containing microsatellites in the control group were analyzed (Figure 5B). These predicted off-target sites demonstrated independent Indel mutations in CRISPR/Cas9. For (CA)_8_(GA)_2_, nine predicted off-target sites contained microsatellites, and one site was randomly selected for further validation using T7ENI cleavage assay (Figure 6A). Results showed that after gel electrophoresis, multiple cut bands can be detected in the selected microsatellite targets even in those in the control group (Figure 6B). Meanwhile, in predicted off-target sites without a microsatellite, a cut band was not detected in the control and genome-edited groups (Figure 6B). Subsequently, for sgRNAs with different lengths, two predicted off-targets containing microsatellites were randomly selected for further validation by T7ENI cleavage assay (Figure S7). Results showed that control groups also detected a high frequency of Indel mutation, explaining that approximately 20% of the background noise in the genome in our study came from microsatellite loci. We also found that when the microsatellite locus was close to the on-target site (unpublished data), it can interfere with the accurate assessment of sgRNA on-target activity using T7ENI cleavage assay and Sanger sequencing.

## DISCUSSION

CRISPR/Cas9-mediated genome editing technology has enabled the accelerated generation of transgenic models, which can promote the rapid development of effective gene therapy strategies. This technology has been applied to the study or treatment of human genetic diseases, including LCA10 and Duchenne's muscular dystrophy 10, 13. Selecting a highly efficient and specific sgRNA is important when conducting CRISPR/Cas9 assay. However, many factors are associated with sgRNA activity and specificity. Various experimental methods can be used to detect sgRNA activity and specificity, and one is to truncate the length of CRISPR sgRNA 30-32. Here, we systematically compared the activity and off-target effects of truncated sgRNA from a standard sgRNA (20-nt sgRNA) in human cell lines. The sensitivity of different methods in detecting sgRNA activity was compared and analyzed. The factors that can affect the accurate detection of sgRNA off-target activity by high-throughput sequencing were also surveyed.

A comparison of the activities of sgRNAs with different lengths targeting the same locus of seven genes or two miRNAs based on T7ENI cleavage and AmpliconSeq assays showed that the effect of length on sgRNA activity was site-dependent. Therefore, when conducting CRISPR/Cas9 experiments, the cleavage activity of sgRNA can be affected by the truncation of 5′-end lengths and may lead to genome editing inactivity. Thus, designing multiple sgRNAs per gene target at a time and selecting one of the most active sgRNAs validated by experiments are necessary. Our results were consistent with a previous study, which reported that sequence length has varying effects on CRISPR/Cas9-mediated gene knockout efficiency 33. As explained in a previous study, the 5′-end lengths of sgRNAs can affect the genome cleavage activity of CRISPR/Cas9, because a conformational checkpoint is present between the DNA binding and cleavage by CRISPR/Cas9, and sgRNA truncation can trap the HNH domain of Cas9 in the checkpoint intermediate with a few mismatches on the DNA 34. This phenomenon may partly explain why the length of sgRNA can affect its activity and specificity.

Target specificity is essential in the development of CRISPR/Cas9 technology. The off-target effect of genome editing is a significant concern for the clinical applications of Cas9. sgRNAs should be designed to maximize their activity and specificity. A number of software that predict and evaluate the off-target effects of sgRNAs are currently available. For instance, sgRNAcas9 and CRISPR-offinder mainly rely on the calculated scores based on mismatches to the sgRNA sequence in CRISPR/Cas9 35, 47. In the present study, *in silico* prediction and TargetSeq were combined, but only a few predicted off-target sites can be verified. On the one hand, the specificity of sgRNAs in different lengths was found to be extremely high based on TargetSeq experiment, and length did not affect sgRNA specificity. On the other hand, the accurate estimation of the off-target effect of sgRNA via sequence similarity searching algorithm is a challenge 35. Therefore, new algorithms that can accurately assess the off-target effects of sgRNAs need to be developed. For instance, the latest studies use machine learning-based predictive modeling to predict CRISPR/Cas9 guide efficiency and specificity 48. The biggest challenge at present is to reduce off-target effects.

Various methods have been developed to detect the activity and off-target effects of sgRNA. In the present study, we compared six different experimental methods used in detecting sgRNA activity. Results showed that AmpliconSeq and TargetSeq detected sgRNA activity with high sensitivity, which can reach up to 0.1%. By contrast, the detection sensitivities of other methods, such as Sanger sequencing of PCR products, *E. coli* TOPO TA cloning and Sanger sequencing, and Sanger DNA sequencing, followed by analysis through TIDE web-based software, were relatively low. Although AmpliconSeq and TargetSeq are generally extremely sensitive and can detect off-target sites that are mutated at a frequency of < 0.1% in a high-throughput manner, strategies based on high-throughput sequencing have limitations, such as sequencing errors, algorithms, probe specificity, capture efficiency, and genomic instability, because CRISPR/Cas9-mediated genome editing depends on the generation of DSBs and subsequent cellular DNA repair process. Therefore, any factor that produces DSB may interfere with the assessment of accuracy of sgRNA activity or specificity. The presence of a microsatellite in the predicted off-target site can produce background noise in the control group, and up to 20% of background noise was due to predicted off-target sites containing microsatellites. Thus, whether other factors can lead to genomic instability and interference with the accurate detection of genome editing specificity require further investigation.

Most importantly, we provided a key molecular feature and analysis strategy to improve the accuracy of detecting CRISPR/Cas9 off-target effects by high-throughput sequencing. Specifically, this study combined *in silico* prediction and TargetSeq to evaluate the specificity of CRISPR sgRNA. Unexpectedly, microsatellites existed in most of the predicted off-target sites for selected sgRNAs. Thus, we first found that when the predicted off-target sites contained microsatellite sequences, these sequences may produce genomic instability or interfere with PCR amplification and sequencing, leading to severe interference in the accurate assessment of off-target effects. Therefore, for the assessment of sgRNA activity or off-target effects, factors that cause genomic instability or interference with PCR amplification and sequencing should be identified and excluded. Taking microsatellites as molecular features, we established a new high-throughput sequencing analysis method, which can significantly improve the accuracy of identifying CRISPR/Cas9 real off-target sites. Our results also suggested that microsatellites interfere with the accurate detection of genome editing on-target activity. Therefore, when designing high-quality CRISPR sgRNA, evaluating if microsatellite sequences are present in the sgRNA target site is recommended.

We systematically evaluated the effects of sequence length and microsatellite on sgRNA off-target effects and compared the activities and specificity of different detection methods for genome editing. TargetSeq combined with *in silico* prediction was well suited for high-throughput assessment of sgRNA activity and specificity. Most importantly, sequence length affected sgRNA activity in a site-dependent manner. After length truncation of sgRNA, the predicted number of off-target sites increased significantly, but the degree of specificity remained unchanged. When analyzing high-throughput sequencing data for genome editing, we need to first check whether a microsatellite sequence is present at on-target or candidate off-target sites.

## MATERIALS and METHODS

### Plasmids

Different sgRNA lengths were designed using CRISPR-offinder to target protein-coding genes and miRNA 35. Oligos (Table S1) for the generation of sgRNA expression plasmids were annealed and cloned into the *Bsa*I sites of pGL3-U6 sgRNA-PGK-Puro vector (#51133, Addgene). Cloned pGL3-U6-sgRNA constructs were sequenced to confirm the correctness of the inserted sequence. CMV-EGFP-hspCas9 vector was used for FACS as described previously 35. Endotoxin-free recombinant plasmids were extracted using Endo-Free Plasmid Mini Kit II (OMEGA).

### Cell culture, transfection, and FACS

HEK293T cells were maintained in Dulbecco's modified eagle's medium (DMEM, Gibco) supplemented with 10% fetal bovine serum (Gibco) and 1% penicillin-streptomycin (Gibco) at 37 °C with 5% CO_2_ incubation. One day before transfection, the cells were trypsinized and seeded into six-well plastic culture plates with DMEM. When cells reached 70%-80% confluency after approximately 24 h, they were transfected with Lipofectamine 2000 (Invitrogen) and Cas9-sgRNA plasmids and were replenished with fresh medium after 6 h of transfection. Then, 48 h after transfection, green fluorescent protein (GFP)-positive cells were sorted using FACSvantage II sorting machine (BD Biosciences, USA).

### T7ENI assay and Sanger sequencing analysis for genomic modification

Harvested or FACS-sorted GFP-positive HEK293T cells after transfection were lysed for gDNA extraction. Using TIANamp Genomic DNA Kit (Tiangen), the genomic region surrounding the CRISPR/Cas9 target site for each gene was PCR-amplified, and PCR products were purified using TaKaRa MiniBEST DNA Fragment Purification Kit (TaKaRa). To detect genome editing-induced mutations, 200 ng PCR products and NEBuffer 2 with ddH_2_O were mixed to a final volume of 19.5 μL and subjected to reannealing as follows: 95 °C for 10 min, 95 °C to 85 °C ramping at -2 °C/s, 85 °C to 25 °C at -0.25 °C/s, and 15 °C held for 2 min. Reannealing was done to enable heteroduplex formation according to previous methods. After reannealing, the products were digested with 0.5 μL of T7ENI at 37 °C for 15 min and analyzed on 2% agarose gel. Afterward, 6× loading buffer (Umibio) with GelRed nucleic acid stain was used to run the DNA. Indel percentage for T7ENI assay was determined by the following equation: 100 × {1 - sqrt [1 (b + c) / (a + b + c)]}, where *a* is the integrated intensity of the uncut PCR product, and *b* and *c* are the integrated intensities of each cut product. Mutated products identified by T7ENI assay were cloned into TA cloning vector and transformed into competent *E. coli* strain. After an overnight culture, colonies were randomly selected and sequenced. The PCR products of sgRNA target site were subjected to Sanger sequencing and further analyzed using the TIDE website (https://tide.nki.nl/). All DNA oligos for constructing sgRNA expression vectors are listed in Table S1.

### AmpliconSeq

AmpliconSeq is based on ultradeep sequencing of PCR products to detect CRISPR-Cas9-induced mutations. Deep sequencing was performed on multiplexed PCR amplicons from gDNA harvested from plasmid transfection of HEK293T cells. Genome sequences, including sgRNA on- and predicted off-target sites, were extracted. The CRISPR cut site was within the first 100 bp of the amplicon (from either the 5′- or 3′-end) to ensure high-quality data. Specific PCR amplicon primers were designed using the NCBI Primer-BLAST tool (https://www.ncbi.nlm.nih.gov/tools/primer-blast/), and product length was set in the range of 200-250 bp. The experiment was performed as follows: (1) PCR amplification of the genomic region that flanks the sgRNA on- or predicted off-target sites for each gene using PrimeSTAR GXL DNA Polymerase (TaKaRa); (2) hybridization mix preparation for adapters P5 (IS1 and IS3) and P7 (IS2 and IS3); (3) blunt end repair of DNA fragment using dNTPs, ATP, T4 polynucleotide kinase, and T4 DNA polymerase; (4) reaction product purification using MinElute PCR Purification Kit (QIAGEN); (5) adapter ligation and fill-in; (6) DNA library amplification by PCR using primer pairs inPE1.0 and inPE2.0 and Illumina multiplex primer; and (7) amplified DNA library sequencing by Illumina Genome Analyzer IIx. All PCR amplicons and library amplification primers are listed in Table S1.

### TargetSeq

Customized next generation sequencing (NGS) target enrichment probes for the capture of targeted regions with an average probe length of 100 bp were designed using AIdesign (available via https://design.igenetech.com). The target probes for TargetSeq assay are listed in Table S2. Genome DNA was isolated using TIANamp Genomic DNA Kit (Tiangen). DNA was assessed using the 2100 Bioanalyzer System. Genome DNA was fragmented to an average size of 150 bp using the Bioruptor^®^ Pico (Bioruptor). Illumina libraries were prepared with the Fast Library Prep Kit (iGeneTech Co., Ltd.). After ligation to NEXTflex DNA barcodes (BIOO Scientific), DNA was amplified using six PCR cycles following TargetSeq^®^ Enrichment Kit specifications. Each library was hybridized to the customized probes following the manufacturer's instructions (iGeneTech™). Captured libraries were enriched using 15 PCR cycles and analyzed via single-end sequencing using the Illumina HiSeq 2500 sequencing platform. The Indel mutations identified by TargetSeq were further confirmed by T7ENI cleavage assay.

### Analysis of genome editing outcomes from deep sequencing data and microsatellites

Sequencing reads were split into individual genomic libraries according to their index read sequences. Genome-editing outcomes from deep sequencing data were analyzed using CRISPRamplicon. Briefly, Indel frequencies were analyzed as follows. First, the quality of raw sequencing reads was checked by FastQC v0.11.3 (http://www.bioinformatics.babraham.ac.uk/projects/fastqc/) and processed by Trimmomatic (v0.35) to trim adapters and low-quality bases and filter low complexity reads (http://hannonlab.cshl.edu/fastx_toolkit/index.html). The presence of adapter sequences or N bases and reads that were > 35 bp after trimming were discarded. Second, qualified reads were aligned to the reference amplicons using BLAST. Finally, the proportion of nonhomologous end-joining outcomes was quantified by CRISPRamplicon. The standalone version of CRISPRamplicon was downloaded from the SourceForge website (https://sourceforge.net/projects/crispramplicon/, unpublished). Subsequently, the microsatellites in given on- or predicted off-target sequences were analyzed using the MicroSatellite identification tool (MISA, http://pgrc.ipk-gatersleben.de/misa/), which can identify and localize perfect and compound microsatellites 49.

## Supplementary Material

Supplementary figures.

Supplementary table S1.

Supplementary table S2.
