# Supplementary material for: Evaluation of the effects of sequence length and microsatellite instability on single-guide RNA activity and specificity
Source: Int J Biol Sci. 2019 Oct 3;15(12):2641–53. doi: 10.7150/ijbs.37152 (PMC6854370; doi:10.7150/ijbs.37152)
Supplement: Supplementary file 1 — Supplementary figures. [file ijbsv15p2641s1.pdf]

Supplementary Tables and Figures

Table S1. Oligos used in this study

Table S2. Targets and probes designed for target capture sequencing

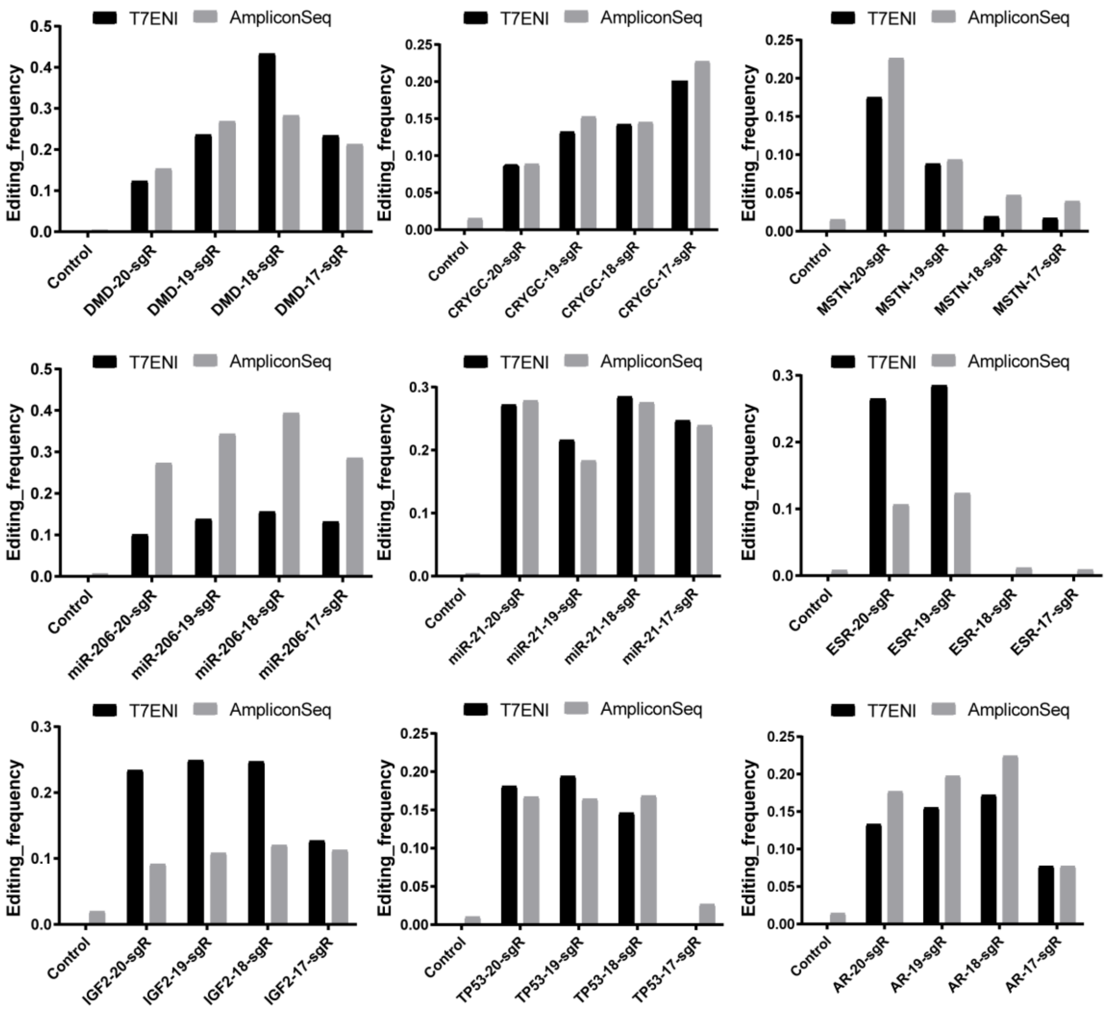

Figure S1. Confirmed activity in different lengths of sgRNAs *via* T7ENI cleavage assay using High-throughput Amplicon Sequencing (AmpliconSeq).

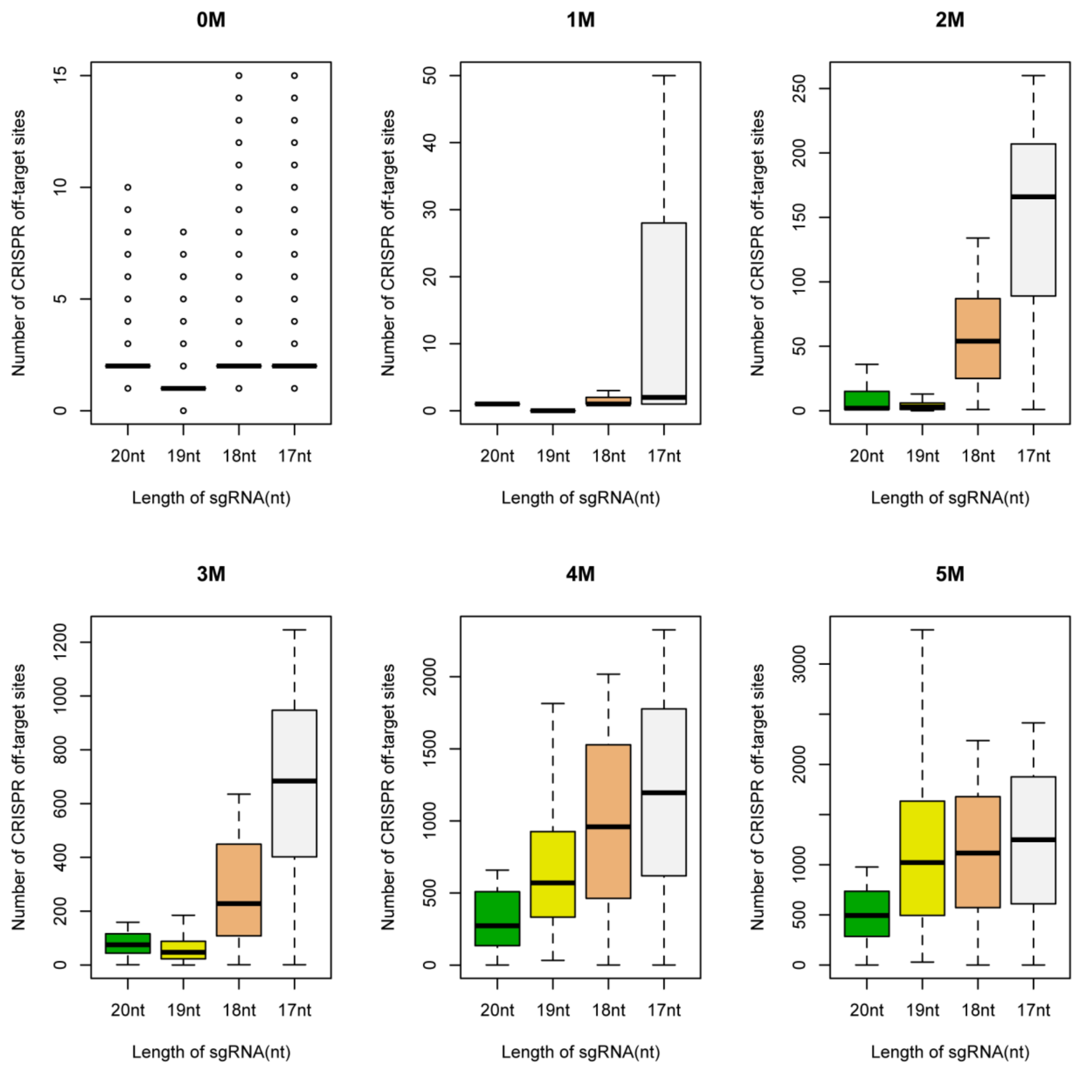

Figure S2. Predicted number of off-target sites with different number of mismatches in different lengths of sgRNAs targeting 100 randomly selected genes. nt: nucleotide; sgRNA: single-guide RNA; M: the number of nucleotide mismatches (1M, 2M, 3M, 4M, or 5M); 0M: the perfect match to the on-target site; if the number of 0M sites > 1, off-target sites could be contained.

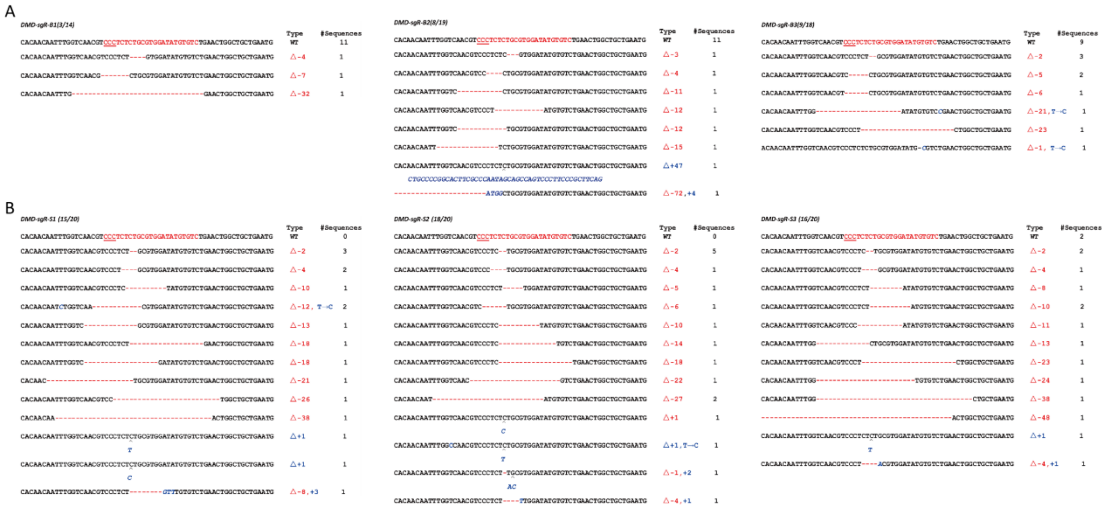

Figure S3. Activity of 20 nt-long sgRNAs targeting *DMD* gene by *E. coli* TOPO TA cloning and Sanger sequencing. “-S” represents sorted cells, and “-B” represents unsorted cells; #sequences is the number of sequencing; Type refers to the Indel mutation type. “---”: deletions; “^”: insertions; “△-number”: deletions; “△+number”: insertions; “(3/14)”: the number of genome-edited targets/number of total sequences; Nucleotides marked in red represents the sgRNA’s on-target site. The experiments were repeated independently three times.

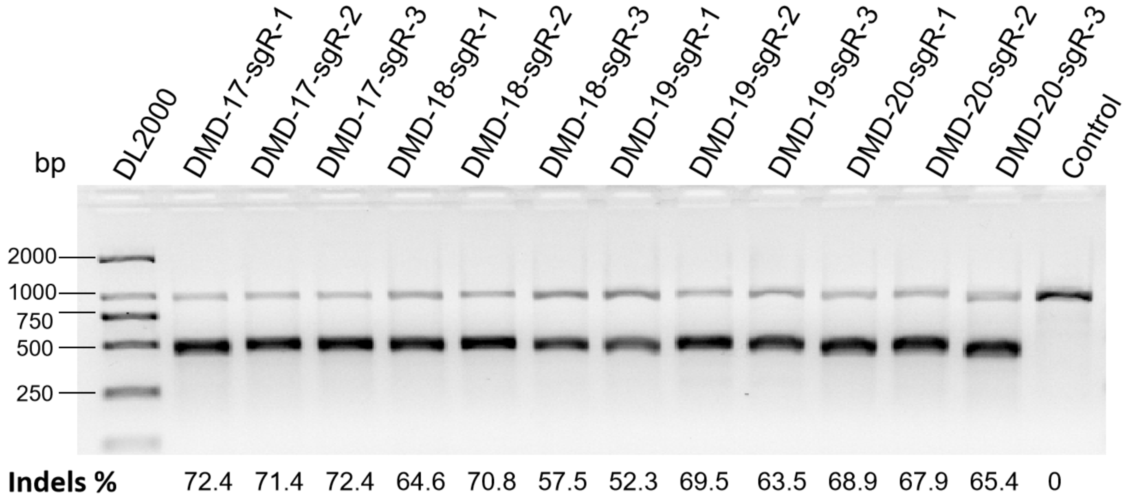

Figure S4. Detection of the activity in different lengths of sgRNA targeting *DMD* gene in the sorted population cells using T7ENI cleavage assay. DL2000: DNA marker; bp: base pair. The experiments were repeated independently three times for each sgRNA.

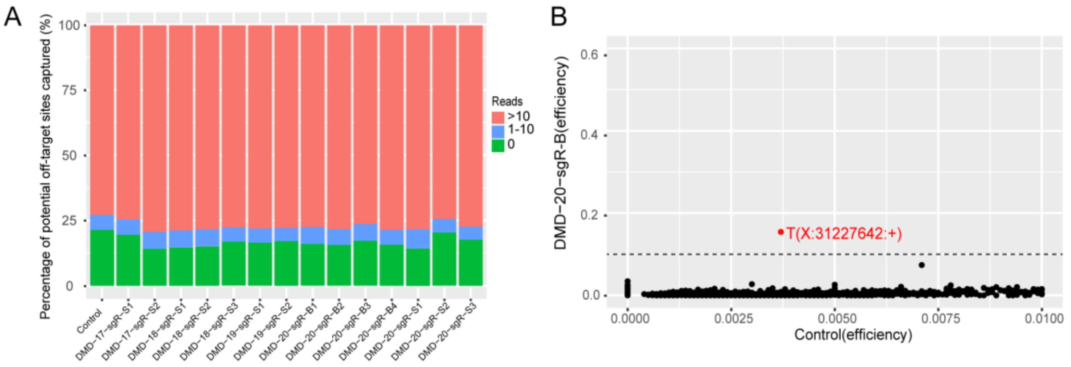

Figure S5. Detection of on- and off-target site cleavage activities for sgRNA targeting *DMD* gene with different lengths by *in silico* prediction and Target capture sequencing (TargetSeq). (A) The percentage of TargetSeq reads captured by the on- and off-target sites for the corresponding locus. The x-axis represents different experimental groups. (B) Detection of the on- and off-target cleavage efficiency of 20 nt-long sgRNAs targeting *DMD* gene by TargetSeq in the unsorted cell population. T (X:31227642:+) represents the on-target site. The x- and y-axis represent the Indel efficiencies of on- and off-targets for sgRNAs in the control and gene-editing groups, respectively. The number of reads ≧10 is the threshold of control group representing the captured target, and the Indel efficiency is ≦1 %.

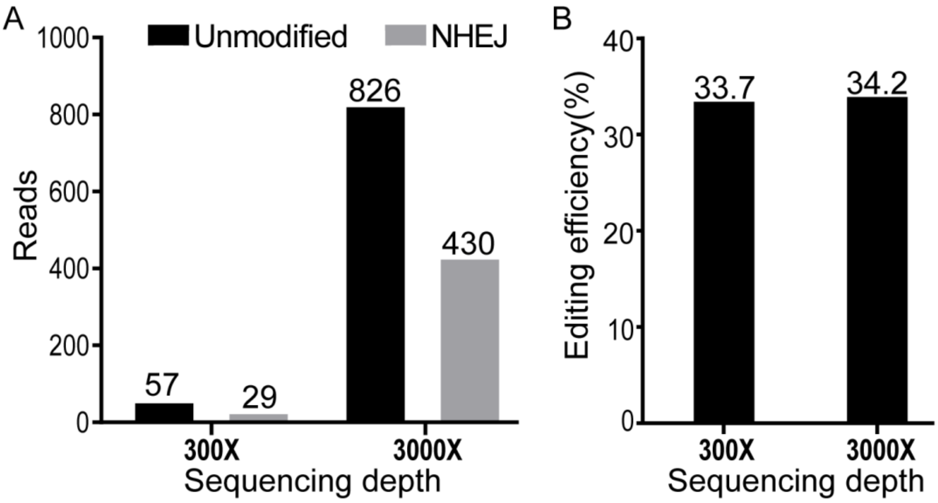

Figure S6. Distinction in the sequencing depth of genome editing efficiency by Target capture sequencing. (A) Number of modified and unedited reads under different sequencing depth conditions. (B) Editing efficiency under different sequencing depth conditions. Unmodified for unmodified sequences; NHEJ: nonhomologous end-joining; 300- or 3000-fold is the sequencing coverage.

A

ID	Sequence(5'-3')
On target	GACACATATCCACGCAGAGAGGG
chr1_178021144 (negative_5)	cACACAcAcaCACaCAGAGAAGG
	ACACAcAcaCACaCAGAGAAGG
	CACAcAcaCACaCAGAGAAGG
	ACAcAcaCACaCAGAGAAGG
chr14_42228686 (positive_5)	cACACAcAcaCACaCAGAGAGAG
	ACACAcAcaCACaCAGAGAGAG
	CACAcAcaCACaCAGAGAGAG
	ACAcAcaCACaCAGAGAGAG

B

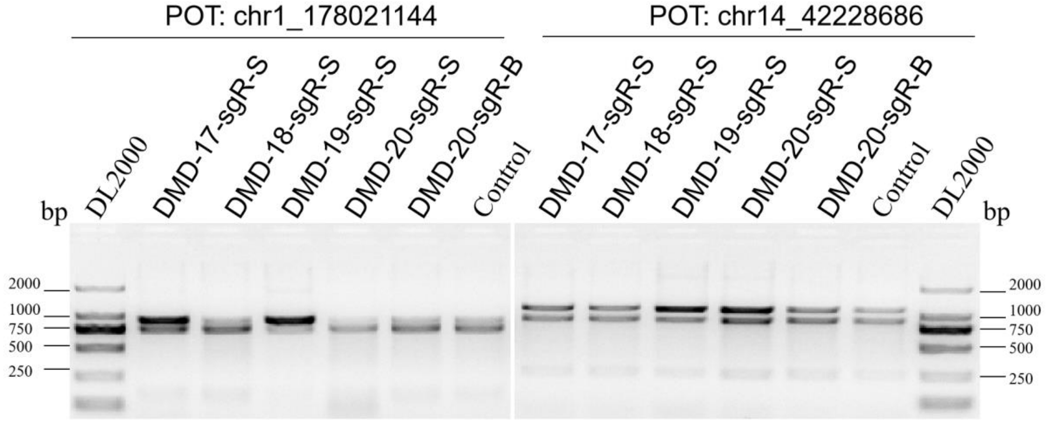

Figure S7. Validation of two selected predicted off-target sites containing microsatellites in 20, 19, 18, and 17 nt sgRNA. (A) Sequence and microsatellite features of potential off-target sites. (B) Detection of cleavage activity of the predicted off-target sites using T7ENI cleavage assay. Control represents negative control group; POT: potential off-target site; NRG: protospacer adjacent motif (PAM);N = A, T, C, or G; R = A or G; DL2000: DNA marker; "-S": sorted cells; "-B": unsorted cells; Nucleotides marked in red and blue colors represent PAM and mismatches, respectively.
